# What next for the Australian Atlas of Healthcare Variation series? Focusing the system on appropriate and sustainable health care

**DOI:** 10.1007/s43999-024-00056-8

**Published:** 2024-12-19

**Authors:** Gillian Giles, Heather Buchan, Carolyn Hullick, Marge Overs, Anne Duggan

**Affiliations:** Australian Commission on Safety and Quality in Health Care, Sydney, NSW Australia

**Keywords:** Unwarranted healthcare variation, Appropriate care, Atlases of healthcare variation

## Abstract

Mapping, identifying and reducing unwarranted healthcare variation is integral to improving the appropriateness of care – minimising wasteful or unnecessary care and redirecting care to those who could benefit most (J Eval Clin Pract 26: 687–696, 2020). The Australian Atlas of Healthcare Variation series has examined variation in healthcare use since 2015. The findings reported in the Atlas series have led to important system changes. National safety and quality standards, mandatory for all hospitals and day procedure services, now require health service organisations to monitor and investigate variation and address unwarranted variation. Clinical care standards have been developed for clinical conditions in which the Atlas series has identified considerable variation. But the overuse of low-value care and underuse of high-value care persists, as suggested by the marked variation the Atlas series continues to uncover. We must now develop an approach that systematically links reporting of data and investigation of variation with a suite of responses to address unwarranted variation. This paper focuses on efforts to reduce low value-care, so that resources can be redirected to supporting high-value care as well as reducing waste and cutting carbon emissions from health care (Med J Aust 216: 67–68, 2022).

## Introduction

The Australian Atlas of Healthcare Variation series (the Atlas) explores the extent to which the use of health care in Australia varies depending on where people live, how their care is funded, their level of socioeconomic disadvantage and, where possible, Indigenous status [1]. Data are age and sex standardised to allow for fair comparisons across local areas. After the national health data agency extracts the data for the Atlas, the Australian Commission on Safety and Quality in Health Care (the Commission) develops interactive maps and graphs, expert commentary and recommendations for action to address unwarranted variation.

The Commission, which is funded jointly by the Australian Government and state and territory governments, has published the Atlas since 2015. As a national body tasked with supporting the health system to improve the quality of health care, the Commission does not have regulatory responsibility for the delivery of health care. In Australia’s federated health system, the Australian Government is mostly responsible for providing primary care and state and territory governments are mostly responsible for providing acute care.

The purpose of the Atlas series is not only to identify variation but to prompt further investigation and action at a local level to reduce any unwarranted variation. The overarching objective is that appropriate care is delivered more often and more consistently.

Although Atlas data do not distinguish between warranted and unwarranted variation in health care, two recurring patterns suggest that healthcare delivery is not matching patient need. Firstly, some groups with the highest burden of disease have the lowest rate of a related investigation or treatment, indicating that barriers to access to care for these groups should be investigated. Secondly, in some areas, there are markedly higher rates of care despite no clear evidence of increased burden of disease in the local population, raising concerns about the degree of benefit gained or potential harms from some healthcare interventions.

While investigating both underuse and overuse of health care is necessary to create a more equitable healthcare system, this article focuses on reducing overuse to minimise the environmental impact of health care.

Based on the pioneering approach of the Dartmouth Atlas in the United States [2], the Commission was prompted to develop the Atlas series after a 2012 study on healthcare variation from the Organisation for Economic Co-operation and Development (OECD) showed Australia’s rates of procedures, including hysterectomy, caesarean section and tonsillectomy, were higher than comparable OECD countries [3].

The Commission has produced four compendium Atlases, published in print and online, covering nearly 90 topics. These large publications have generated considerable media interest, and the findings have been picked up by policy makers and incorporated into national agreements [4], policies [5] and safety and quality standards [6]. However, the risk of publishing large compendiums is the diffusion of focus from a vast array of clinical topics presented at one time. For this reason, the reporting strategy has evolved to producing themed reports on key clinical topics, such as the dispensing of opioid [7] and antipsychotic medicines [8]. These time series reports, which identify local areas with consistently high or low rates, have enabled a greater focus on engagement with local areas after publication.

### The grey zone: what is low-value care?

Low-value care describes interventions where evidence shows little or no benefit for particular patients, or the risks outweigh the expected benefits [9]. Determining the value or appropriateness of care is not clear cut. Evidence on the benefits and harm of interventions is not always generalisable to all patients and may not align with patients’ needs [10]. The value of care is on a continuum, from appropriate for all at one end to inappropriate for all at the other [10]. Most care falls into the grey middle zone, where it is appropriate in different cases [10, 11].

Reducing low-value care and increasing high-value care (which includes timely diagnosis and effective treatment) will benefit both patients and the health system. A focus on appropriate care – care that will give the best outcome, according to available evidence and the patient’s goals – is key to creating a more sustainable health system.

### The Atlas model: embedded and engaged with the health system

Analyses of data to generate reports about variation have limited impact on reducing unwarranted variation in health care [12]. Atlases provide important information about geographical variation in health care, but they need to be part of a larger program of system change to improve care [13]. In recognition of these limitations, the Atlas program is designed to engage with the health system to drive change. Consulting with government, clinicians and clinical organisations to select topics for investigation and interpret findings creates influential champions who are essential to reaching the primary audiences: policy makers, clinicians and health service organisations who can act on the data to improve care [13].

As well as providing data and commentary, we respond to findings by presenting recommendations for action using multiple system levers. Atlas data reach and influence government agencies and health departments, health service organisations and providers, clinicians and clinical colleges across Australia. The Atlas’s home at the Commission not only provides this deep reach into government, it allows insights into the system and avenues to address unwarranted variation. The Atlas complements other Commission programs focused on improving appropriateness of care − such as safety and quality standards, clinical care standards and partnering with consumers − amplifying the effects of all. The Commission does not provide health care, so we seek to use the levers available to prompt investigation and action for those who do − at a federal, state, territory and local level.

### Impact by design

**Fig. 1:**
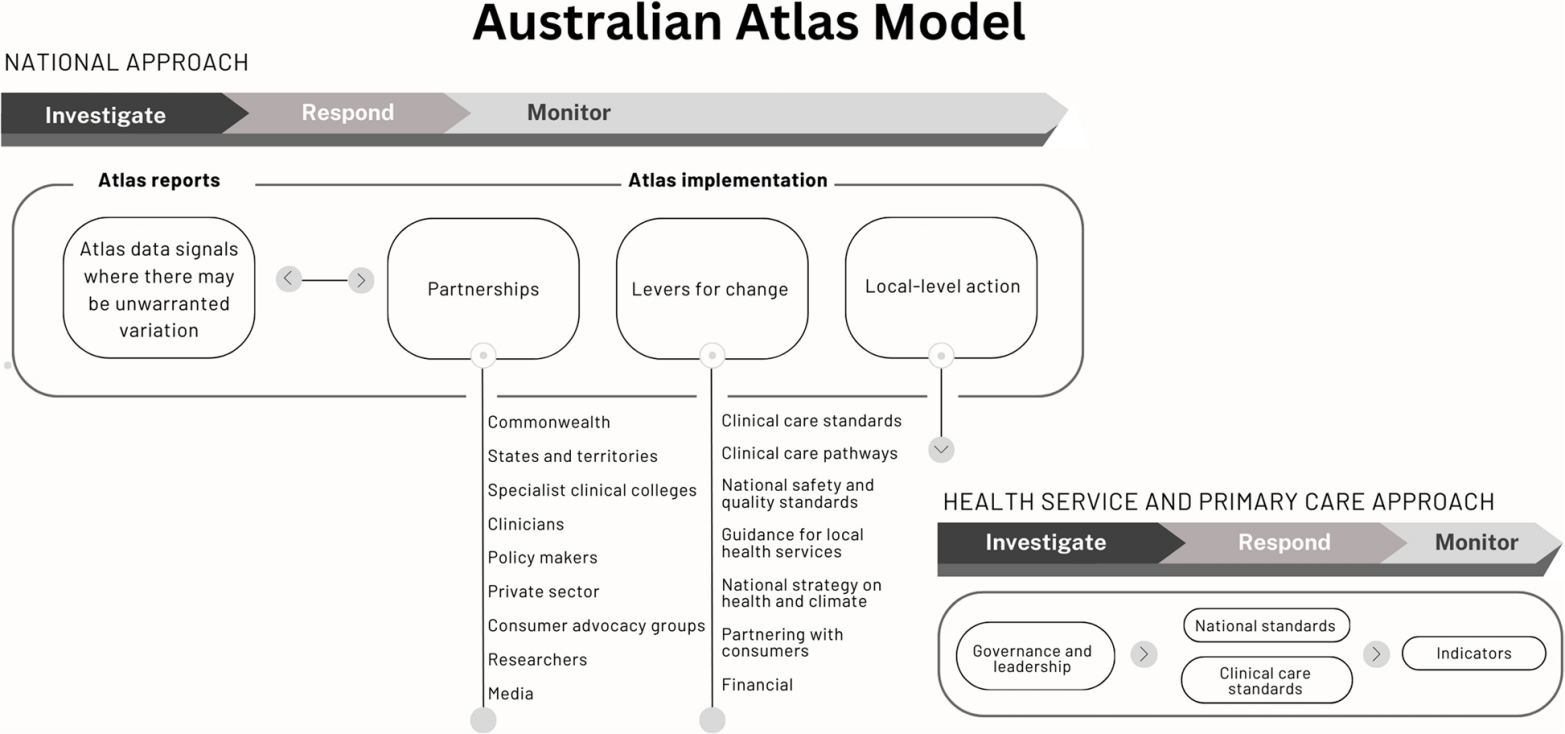
Australian Atlas model

The Atlas model involves three key elements to ensure reach and influence: partnerships, levers for change and local-level action (Fig. 1).

#### Partnerships

We collaborate with high-profile contributors and topic experts when selecting indicators and writing content for the Atlas series. Project governance incorporates representatives from all states and territories, the private sector, primary and acute care sectors, specialist medical colleges and other clinical colleges, and consumer advocacy groups. Typically, we consult with more than 150 stakeholders when developing each Atlas. Atlas findings are presented in tailored reports, briefings and meetings with local areas in which there is consistently high variation.

#### Levers for change

In a federated health system such as Australia, gaining consensus on priority areas for improvement is challenging. Systems thinking underpins our approach. Recognising that health service organisations are, and operate within, complex systems [14], we need to consider how different levels of the health system interconnect. Individual interventions such as policy interventions or audit and feedback work some of the time, but none works all the time [15]. Strategies to improve the appropriateness of care must acknowledge the interrelationship between parts of the system [16].

The Commission has used levers for change in a variety of ways across the system. Changes in the health system are usually the result of the accumulated efforts of many players over many years, rather than a single intervention. The following examples describe the levers the Commission has used with the aim of influencing the way health care is delivered at the point of care.

##### Clinical care standards

The Commission’s clinical care standards contain a small number of evidence-based quality statements that describe the clinical care a patient should be offered for a specific clinical condition or procedure and indicators for health service organisations to monitor implementation. Clinical care standards are different to clinical guidelines – they do not detail all aspects of treatment and care for a specific condition but instead focus on priority areas for improvement based on clinical guidelines and best evidence. The Commission has developed clinical care standards for conditions and procedures for which the Atlas has shown considerable variation, for example cataract surgery [17], colonoscopy [18], hip fracture [19] and osteoarthritis of the knee [20].

##### Clinical care pathways

Clinical care standards developed as a result of Atlas data have been incorporated into web-based portals (known as HealthPathways) that guide primary healthcare providers, particularly general practitioners, on local referral pathways and evidence-based treatments [21].

##### National safety and quality standards

The value of monitoring variation is reflected in the National Safety and Quality Health Service (NSQHS) Standards, which are mandatory for hospitals and day procedure services [6]. Developed by the Commission, the standards aim to protect the public from harm and to improve the quality of health service provision. As a component of clinical governance, the NSQHS standards require health service organisations to measure and monitor variation to identify unwarranted variation, and to regularly review and improve the appropriateness of clinical care.

##### Guidance for reviewing healthcare variation at a local level

Audit and feedback can reduce unwarranted healthcare variation when there is engagement with clinicians, meaningful indicators, a clear improvement plan and respect for clinical expertise [22]. The Commission has produced a user guide that presents a practical six-step approach to reviewing clinical variation data and case studies that put those steps into action [23]. The user guide provides a systematic approach to identifying and addressing unwarranted variation including the use of audits, feedback and case studies showcasing best practice and innovation.

##### National strategy on health and climate

The Commission collaborated with the Australian Government to develop recommendations in the National Health and Climate Strategy [24] to reduce carbon emissions by tackling unwarranted variation and providing appropriate care. The Strategy recognises that unwarranted variation (low-value care) contributes to carbon emissions. The recommendations are designed to equip health service organisation boards, leadership teams and clinicians to shift resources from lower-value to higher-value activity and to monitor and evaluate systems for improving appropriateness of care.

##### Partnering with consumers

When patients are better informed about the benefits and harms of interventions, they are more likely to decline low-value care [25]. In response to Atlas findings, the Commission developed guidance on shared decision-making and decision-support tools for osteoarthritis of the knee and antibiotic use for middle ear infection [26].

##### Financial

The Atlas found high rates and substantial variation in potentially preventable hospitalisations for chronic disease [27]. To address fragmented care, the Commission recommended alternative approaches to funding models, which led to work with the national funding agency and pricing authority on payment models to incentivise integrated care. Atlas findings have also led to the Australian Government making changes in funding for colonoscopy. Concerns about overuse, with higher rates in major cities than remote areas and higher rates in areas of socioeconomic advantage, led to changes in financial rebates designed to ensure appropriate intervals between colonoscopies [28].

#### Local-level action

The Atlas model is not a prescriptive approach to addressing unwarranted variation because locally crafted and targeted approaches are needed. As the NHS Atlas series recognised, the reason for sharing data on variation is to prompt further investigation of variation and actions to address unwarranted variation, not to define what is right or wrong [13].

With many factors contributing to unwarranted variation, a combination of national, state and territory, and local approaches is needed to improve the appropriateness of care and effectively reduce low-value care [15].

At a local level, every hospital in Australia must be accredited to NSQHS Standards. Implementation of the Standards includes the requirement for leadership teams and clinicians to investigate, respond to and monitor unwarranted variation. This approach recognises that overuse and underuse may coexist and that local services are best placed to distinguish between the two.

An example of a local approach is a large health service district in Sydney that embedded a focus on healthcare variation within its governance structures by establishing a healthcare variation executive steering committee. Comprised of department heads and safety and quality leads, the committee reports to the board and acts as a conduit between the board, leadership team and clinical leaders for systems improvement.

Following the publication of Atlas reports, we engage with local leadership teams and clinicians to investigate, respond to and monitor unwarranted variation. For example, we have worked with primary care organisations with consistently high dispensing rates of high-risk medicines such as opioid analgesics and antipsychotic medicines. We provided tailored reports and briefings to assist them to understand the data and identify unwarranted variation.

## Atlas impact: data to action

### How variation data prompted clinical standards on heavy menstrual bleeding

The First and Second Atlases showed considerable geographical variation in the rates of endometrial ablation and hysterectomy for women with benign conditions, such as heavy menstrual bleeding [27]. In response, the Commission developed a clinical care standard designed to ensure that women with heavy menstrual bleeding are offered the least invasive and most effective treatment appropriate to their clinical need [29]. Using the Atlas data and the clinical care standard, health services have the tools to identify and address unwarranted variation in care and ensure women with heavy menstrual bleeding are supported to make informed choices about the care that is right for them [30]. For example, Atlas data showing high hysterectomy rates in a regional area prompted a gynaecologist to review whether care offered to women with heavy menstrual bleeding aligned with the clinical care standard. The gynaecologist went on to develop a model of care that fast-tracked medical management for women with heavy menstrual bleeding [31].

Seven years after the Second Atlas, the Commission published time series data to determine where progress has been made and to identify areas with consistently high rates [32]. The report found a 20% decrease in the national hysterectomy rate over eight years. The Commission published the revised Heavy Menstrual Bleeding Clinical Care Standard [33] at the same time as the Atlas time series data. The combination of the time series data and the clinical care standard has sustained focus on the appropriate treatment of heavy menstrual bleeding.

### Reducing variation in knee arthroscopy rates

Australia recorded high rates of knee arthroscopy in the 2000s [3] despite mounting evidence of no benefit for most people with knee ostearthritis [34, 35]. In 2015, the first Atlas also reported high rates of knee arthroscopy and seven-fold variation across Australia for people aged 55 years and over [1].

To address variation, the Commission:

• Referred findings to a taskforce that reviews government funding of health services.
• Released the Osteoarthritis of the Knee Clinical Care Standard to support clinicians to provide evidence-based care for knee osteoarthritis [20].
• Engaged with a health department in a state with high rates of knee arthroscopy by briefing the leadership team and preparing a tailored report.

In 2018, the Australian Government revised the Medicare Benefits Schedule (MBS) item numbers for the procedure, and removed funding for knee arthroscopy for degenerative changes [5]. Around the same time, clinical recommendations highlighting the lack of benefit of knee arthroscopy for osteoarthritis helped to drive rates down [36]. Internal Commission analysis of MBS data showed that, in the 10 years from 2013 to 2023, Australia’s rate of knee arthroscopies for people aged 55 years and over had more than halved (from 495 to 209 knee arthroscopies per 100,000 people).

## Challenges and limitations

### Impact of COVID-19

Health systems are under enormous pressure following the COVID-19 pandemic. Budget constraints and workforce shortages threaten the safety and quality of healthcare delivery. The challenge is to cut through the myriad competing priorities so that the benefits of monitoring healthcare variation and responding to unwarranted variation are clear.

#### Use of linked data

An issue with collection of health data in Australia is that information about patients’ health care is split across multiple datasets held and managed by different governments. Tracking experiences across these data divides would provide a more informative picture of healthcare quality, but this is not always straightforward. To date, it has not been possible to easily access timely linked data at a national level, and there are inconsistencies and limitations across federal, state and territory datasets. Recent changes in intergovernmental arrangements are expected to streamline access and coverage, and so the use of linked data assets might be feasible for future reporting in the Atlas series, allowing examination of the outcomes of care provided.

#### Inability to report at health service level

The Atlas series relies on nationally consistent administrative data, with analysis by where the patient lives rather than where the care was provided. The findings cannot be directly attributed to the performance of a health service organisation or local model of care. By not reporting at a health service organisation level, we are not harnessing a lever for change – reputational incentives [37, 38]. Well-designed systems of public reporting can improve performance of health service organisations [39].

#### Difficulties in identifying unwarranted variation

Local interrogation of data and investigation is necessary for health service organisations to frame their own lines of inquiry. Identification of variation is only the first step – determining whether variation signals overuse, underuse or misuse is less straightforward. We need to improve the way we distinguish and signal unwarranted variation [40, 41].

## New directions

### Targeting low-value care for sustainability

The global imperative to reduce health system contributions to climate change provides a powerful incentive to reduce unwarranted variation. Around 30% of health care is estimated to be waste, duplication or of low value [42]. Ensuring appropriate care and avoiding unnecessary investigations and treatment are therefore central to improving the sustainability of health systems [41].

The purpose of the Atlas series is not only to improve the appropriateness of health care; we must also consider the pressing problem of overuse and the impact of misdirecting limited resources towards health care of little value. Sustainable health systems need to identify and eliminate avoidable waste, minimise low-value care that misallocates scarce resources, and ensure activity is focused on high-value care. We can position the Atlas series as a tool to direct health systems to provide high-value care by better describing how unwarranted variation can signal low-value care [43].

Efforts to reduce the carbon footprint of the health system require a nuanced consideration of both the under- and overuse of health care. As well as reducing waste and low-value care, we recognise the need to reduce healthcare demand by addressing the socioeconomic determinants of health [41].

#### Engaging all levels of the system in addressing low-value care

A systematic approach is required to address the many drivers of inappropriate care [44]. Health service board members, leadership teams and clinicians all need to be engaged, including executive support of clinicians to address low-value care [37].

Healthcare variation data can only be used to guide quality improvement when paired with local insights. Health service organisations therefore need robust systems to interrogate their data and distinguish warranted from unwarranted variation. These systems will inform the decisions made by boards and leadership teams to better target interventions and allocate resources more effectively to improve the quality of care [45].

At a health service organisation level, robust clinical governance is needed to ensure healthcare variation data are used to improve care. The Commission is increasing its focus on clinical governance – to better support boards and leadership teams to manage clinical as well as corporate risks, with a focus on delivering appropriate health care. Reducing unwarranted variation is a responsibility for boards and leadership teams and this has been reflected in the clinical governance actions in the National Safety and Quality Health Service Standards [6]. This responsibility has also been reflected in the National Health and Climate Strategy’s recommendation for boards and leadership teams to address unwarranted variation in line with their organisation’s risk management framework [24].

A key element of clinical governance is creating a culture of resource stewardship to redirect investment in wasted resources to higher-value interventions [38]. There is an opportunity to also frame resource stewardship in the context of improving environmental sustainability [46].

Changes in culture and leadership are more important than measurement alone. Creating a culture of resource stewardship requires clear communication:

• To boards and clinical leadership teams, with reports on unwarranted variation in quality and utilisation rates [47].
• From boards and leadership teams, to set expectations prioritising the importance of reducing unwarranted variation [48].

## Conclusion

The Australian Atlas series has already shown the impact of shining a light on healthcare variation. While it is important to continue to publish reports that map healthcare variation, particularly time series data, we need to better support the system to respond to overuse, underuse and misuse of health care.

With a global imperative to focus the health system on sustainability, a continued focus on the examination of healthcare variation can direct improvement efforts. The effectiveness of the Australian Atlas series is contingent on deep engagement across the health system. The challenge now is to harness these partnerships to change the culture and also empower leadership teams and clinical champions to create a more appropriate and sustainable system.

## Data Availability

The dataset analysed during the current study is available from the corresponding author on reasonable request.
